# Utilization of health services in relation to mental health problems in adolescents: A population based survey

**DOI:** 10.1186/1471-2458-6-34

**Published:** 2006-02-16

**Authors:** Henrik D Zachrisson, Kjetil Rödje, Arnstein Mykletun

**Affiliations:** 1 Norwegian Institute of Public Health, Div. Mental Health, P.O. Box 4404, 0403, Nydalen, Oslo; 2 Norwegian Institute of Public Health, Div. Epidemiology, Dept. Health Statistics, P.O. Box 4404, 0403, Nydalen, Oslo; 3 Action for Health Research, Simon Fraser University, 8888 University Drive, Burnaby, BC, V5A 1S6, Canada; 4 Research Centre for Health Promotion, University of Bergen, Christiesgt.13, 5015 Bergen, Norway

## Abstract

**Background:**

Only a minority of adolescents reporting symptoms above case-levels on screenings for mental health seeks and receives help from specialist health services. The objective of this study was to a) examine help-seeking for symptoms of anxiety and depression in relation to symptom load dimensionally, b) identify the level of specialization in mental health among service-providers, and c) identify associations between mental health problems and contact with different types of health services.

**Methods:**

This cross-sectional school-based study (response-rate 88%, *n *= 11154) is based on Norwegian health surveys among 15 and 16 year olds.

**Results:**

We found a dose-response association between symptom-load and help seeking. Only 34% of individuals with mental symptom-load above 99^th ^percentile reported help-seeking in the last 12 months. Forty percent of help seekers were in contact with specialists (psychiatrists or psychologists), the remaining were mainly in contact with GPs. Mental health problems increased help seeking to all twelve service providers examined.

**Conclusion:**

It might not be reasonable to argue that all adolescents with case-level mental health problems are in need of treatment. However, concerning the 99^th ^percentile, claiming treatment need is less controversial. Even in the Norwegian context where mental health services are relatively available and free of charge, help-seeking in individuals with the highest symptom-loads is still low. Most help seekers achieved contact with health care providers, half of them at a non specialized level. Our results suggest that adolescents' recognition of mental health problems or intention to seek help for these are the major "filters" restricting treatment.

## Background

The prevalence of mental health problems in adolescent populations is found to be between 16% and 22% across several studies [1-7]. Within the EU and US, the finding that a minority (between 13% and 36%) of adolescents with mental health problems seeks professional help seems robust [4,6,8]. These estimates are however suspected to be strongly biased from use of different measures of mental health and cut-offs, and are also expected to vary with availability and acceptability of the services.

It is commonly presumed that the help-seeking pathway passes through several "filters", each of which exclude some help-seekers from the process [9,10]. This model has been adjusted for adolescents by Zwaanswijk and colleagues [11]. The first of these filters is symptom recognition by the adolescent, the second GP's recognition of symptoms in the patients, the third being GP's decision of treatment or referral to specialist services. The model fits with the Norwegian health-care system, where all contact is supposed to go through the GP. This much used model focuses on the importance of generalist health services in help-seeking for mental health problems. In spite of this model, studies addressing help-seeking for mental health problems among adolescents often maintain focus on specialist mental health services, and thus ignore the importance of the first "filters". For instance, one American study report the proportion of adolescents seeking help for mental health problems from specialists being about 35% [4]. European studies seem to report somewhat lower rates. A French study found that among adolescents with probable psychiatric problems, 14% had consulted a mental health professional during the last year [12]. Two Dutch studies found that among adolescents with mental health problems, the percentages referred for mental health services within the past year were 13% [6] and 8% [8] respectively. The importance of generalist health services as a "filter" for help-seekers with mental health problems has been supported by other studies. Findings suggest that among adolescents reporting mental health problems, the use of mental health services was associated with use of other health services [12,13]. This impression is supported by Cohen and colleagues [14] who found that when teenagers' emotional and behavioural problems were discussed with physicians it was generally done so in the context of physical health problems. The few population based studies have addressed adolescent help-seeking directed towards the general health-services, and found rates varying from 13% to 25% [1,15,16]. Mental health problems are reported to increase help seeking from all health services [15]. This finding was however not controlled for somatic complaints.

Several issues are still in need of further investigation. First, variations of pathology within the syndromal area as well as issues of sub-syndromality require these case-level approaches to be supplemented by a dimensional approach. Second, the degree of mental health specialization of health-providers which adolescents reach for seeking help is not answered from the current literature. Third, as a consequence of the finding that mental health problems often are discussed with GPs in the context of physical health complaints, we aim at examining whether mental health problems increase help-seeking to general practitioners (and other providers of health care services) beyond help-seeking that follows from comorbid somatic conditions and psychosomatic symptoms. The current study aims at targeting these three questions employing a large population study of 15–16 year old Norwegians.

## Methods

### Design and participants

The materials used in this study were collected from a cross-sectional health survey among pupils in 10^th ^grade (15–16 year old) in both urban and rural regions of Norway, including Oslo, and the counties of Hedmark and Oppland in the southeastern part of Norway. The Norwegian Institute of Public Health was in charge of conducting the data collection.

All 10^th ^grade school classes in the selected counties were invited to participate in the study. This is the last year of obligatory schooling in Norway.

Participation in this health survey was voluntary and pupils gave their written informed consent for using the data for research purposes before filling out the questionnaires. Parents also had the option of withdrawing their child from the study.

Questionnaires were filled out during school hours in the classrooms. Information about the survey and instructions on how to complete the questionnaire was given in the classrooms by specially trained field workers who were also responsible for gathering the completed questionnaires.

Those students not present were given the questionnaire to be answered later. Students still not responding were mailed the questionnaire at home to be answered and returned in an already stamped and addressed envelope. The final response rate was 88% and the total number of respondents was 11154.

The study protocol was approved by the Regional Comity for Medical Research Ethics and by the Norwegian Data Inspectorate.

### Measures

#### Mental health status

Mental health status was measured using the Hopkins Symptom Check List, 10 item version (HSCL-10), which is a condensed version of the HSCL-25 [17,18]. The HSCL-10 asks for presence of symptoms during the last week, and includes 3 questions of symptoms of anxiety, 6 questions of depression, and one question relevant for both anxiety and depression. Responses are encoded on a four-point Likert scale from "not troubled" to "heavily troubled". For the purpose of the first aim (dimensionality in symptom load in relation to help-seeking), we apply the mean-score of the scale as a continuous variable (each sum-score value labelled with percentiles in the total population). For the last aim, we apply the conventional dichotomy for case-level symptom load with cut-off at and above 1.85 on the mean-score (range 1–4).

#### Help-seeking for mental health problems

Participants were asked whether they during the last 12 months had "had a mental health problem for which help had been sought". Response categories were "yes" or "no".

#### Use of health services

Separate from the question on help-seeking for mental health problems cited above, the pupils were asked about their use of various health services during the last 12 months. They were given a list including school health services, youth health clinic, adolescent pedagogical psychological service, psychologist or psychiatrist, family counselling, other medical specialist, emergency ward or outpatient clinic, hospital inpatient, municipal social service agency, physiotherapist, and alternative therapy. For each of these the pupils were asked whether they had used this health service; none, 1–3 times, or 4 or more times during the last 12 months. The content and quality of the contact with health service providers could not be determined from the questionnaire. The response categories 1–3 times and 4 or more times were therefore collapsed into one category for analyses.

#### Hierarchical arrangement of services specializing in mental health problems

Adolescents commonly reported contact with multiple service providers. In order to examine degree of mental health specialization in the contact adolescents achieved, we defined a hierarchical ranking of level of specialization. At the lowest level, we defined services mainly provided by nurses or other health-providers without academic degree or special training in mental health problems. On the next level, we defined all MDs regardless of specialization, psychiatrists excluded. The GP was not addressed as a separate level because referral to specialist services is done by the GP. Consequently, most adolescents attending other medical specialists would have been examined by the GP. The third level comprised pedagogical-psychological services and family counselling, where most providers have some specialisation in mental health, as they commonly handle behavioural problems. The fourth level included clinical psychologist and psychiatrist, regarded as the health care providers with the most advanced training in mental health problems. Adolescents with multiple contacts were in this hierarchal arrangement encoded as to their most specialized health-care provider contacted.

#### Somatic and psychosomatic symptoms

Somatic and psychosomatic conditions were defined as report of having had asthma, allergy, eczema, or within the last 12 months having had inflammation of the ear, soar throat, bronchitis, lung inflammation head ache, or pains in shoulder, neck, legs, knees, stomach, or back. Response categories were "yes" or "no".

### Statistical analysis

The dimensional analysis of associations between symptom load (HSCL-10 score) and having a mental health problem for which help had been sought during the last 12 months, was illustrated graphically with proportions (with 95% confidence intervals [CI]) having sought help for each symptom load. The second analysis concerned the degree of specialization in health services contacted by adolescents who report help-seeking for mental health problems, and the comparison of this with those who not report such help-seeking. For this purpose we applied cross-tabulations. In our third analysis, we attempted to disentangle help-seeking for mental health problems from somatic and psychosomatic conditions in adolescents' help-seeking towards each of the different health services listed. For this purpose, we applied logistic regression analyses with help-seeking toward each of the help-providers listed as dependent variable and case-level mental health problems as independent variable. Associations between mental health problems and help-seeking were reported as odds ratios (OR) with 95% CI with and without adjustment for somatic psychosomatic conditions. SPSS version 11.5 was used for all analyses.

## Results

Seven hundred and twenty seven adolescents (6.9%) reported that help for mental problems had been sought during the preceding 12 months. Non-responders to this particular question (N = 683, 6.1%) reported a slightly higher number of contacts with general health services (also psychologist and psychiatrist) and also slightly higher symptom level of anxiety and depression than individuals not having sought help for mental health problem. These individuals were excluded from further analyses including this question.

**Figure 1:**
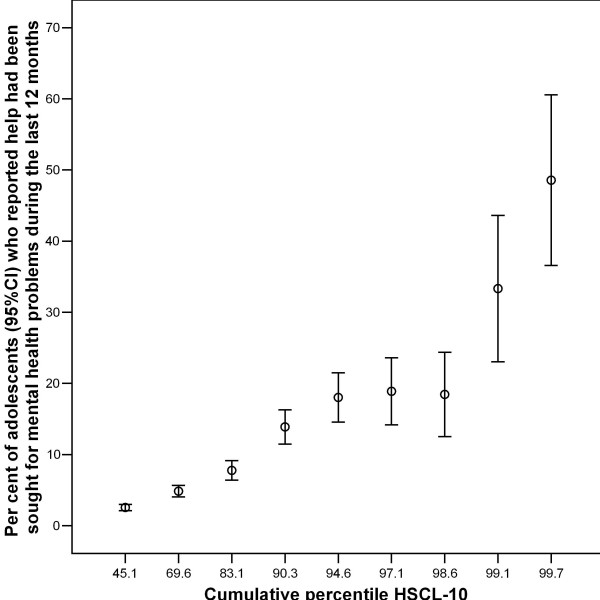
Mental health symptom load (cumulative percentile on the HSCL-10) in relation to per cent of adolescents seeking help for mental health problems.

There was a dose-response association between symptom level of anxiety and depression and having had a mental health problem for which help had been sought during the last 12 months (*p *< .001, Fig 1). The association was however not very strong (Nagelkerke R-square 0.12). Among individuals within the highest percentile on anxiety and depression, less than half (34%) reported help-seeking for mental health problems last 12 months. Within the highest 10^th ^percentile 24% of adolescents reported seeking help for mental health problems.

Table 1 presents a cross tabulation between help-seeking for mental health problems and a hierarchical arrangement of health services according to their specialization in mental health. Of adolescents reporting that help for mental health problems had been sought during the last 12 months, 40% achieved contact with a clinical psychologist or a psychiatrist. Further 8% were in contact with psychological-pedagogical services or family counseling as their most specialized contact. The largest group (44%) was in contacts with MDs (excluding psychiatrists) as their most specialized contact within the mental health services. A minority (3%) had contact with the lest specialized level (nurses, etc.) only, and 4% reported no contact with any of the above mentioned services despite report of help for mental problems being sought in the same period.

Finally, we examined if case-level anxiety and depression cross-sectionally predicted contact with health care providers with and without adjustment for comorbid somatic conditions and psychosomatic symptoms (table 2). Separate logistic regression models were performed for contact with each health-care provider, and results are presented according to the strength of the association. Case-level anxiety and depression increased help-seeking for all services. Strongest effects were found for providers conventionally associated with mental health care (e.g. psychiatrist and clinical psychologists), and accordingly, weakest effects were found for providers commonly not dealing with mental disorders (e.g. physiotherapists). The effects of case-level anxiety and depression on help-seeking was partly explained by adjustment for somatic conditions, and this attenuation of effect was found to be stronger in health-care providers commonly not dealing with mental health problems, e.g. physiotherapists. Case-level anxiety and depression was a weak predictor of contact with GPs (relative to effects of mental symptoms on other help-providers), and most of this effect was explained by adjustment for somatic conditions and psychosomatic symptoms.

## Discussion

There are five main findings of this study: (1) We found a dose-response association between symptom load of anxiety and depression and help-seeking for mental health problems, which is contrary to the common use of case-levels in such studies. (2) The rate of help-seeking for mental health problems (previous 12 months) in the most severe percentile of anxiety and depression was only 34%. (3) Further, we found that mental health problems increased help-seeking from all health-service providers, also providers without special competence in the mental health area. (4) MDs without special training in treatment of mental health problems had the highest number of consultations with adolescents with such problems. There was, however, hardly any effect of mental health problems on MD consultation having adjusted for somatic health complaints. (5) Most adolescents who reported having sought help for mental health problems reached a MD or better qualified professional helper, and rather few saw no health provider at all, or nurses without special training only.

The major strength of this study is the population based design with good participation rate, which helps avoiding biases commonly found in studies in clinical contexts. Probably the most important limitation of our study is the use of HSCL-10, which limits the screening to symptoms of anxiety and depression. Externalizing mental disorders like ADHD, alcohol problems, drugs, and other behavioural problems are not covered by this mental health screening. Externalizing problems might be the reason for help-seeking for mental disorders in individuals with low levels of anxiety and depression, but not low help-seeking in individuals with high levels of anxiety and depression. Symptoms of anxiety and depression are measured with a rather short self-report questionnaire, asking for the presence of symptoms during the last week only. This implies imperfect sensitivity and specificity to these conditions compared with diagnostic interviews. Problems of reliability might contribute to underestimation of the strength of the association between mental health problems and help-seeking. The content of the contact with health care providers is not specified, contributing to overestimation of the association between mental health problems and help-seeking. The likely effect of these limitations is however unclear. Further limitation stems from the cross-sectional design: Help-seeking can only be reported in retrospect, and symptoms of anxiety and depression only at present. This limitation might contribute to explain help-seeking for mental health problems in individuals without current symptoms.

The examination of symptoms of anxiety and depression as continuous variables hampers comparison with previous research which applies cut-offs for case-level, commonly based on self-report questionnaires, or in fewer cases on clinical interviews. If we apply a prevalence rate of mental health problems among adolescents comparable to those reported in other studies, 16% of the highest 17^th ^percentile on the HSCL-10 has sought help during the last 12 months. In line with recent developments within this area in adults [19], we do not argue that everyone with a case-level symptom load (or a mental diagnosis) is in need of professional help. We do, however, argue that individuals above the 99^th ^percentile should receive professional attention, and at least in this respect, our study has revealed under-treatment of mental health problems among adolescents.

We are the first to report a dose-response association between mental health problems among adolescents and help-seeking for this. The more mental health symptoms, the more adolescents seek help. However, 76% of adolescents with symptom load above the 90^th ^percentile and 66% above the 99^th ^percentile still did not seek help. In other words, the vast majority of adolescents with severe symptoms of anxiety and depressions do not seek help for these problems. This is in accordance with the model of Goldberg [9], describing the patient's recognition of symptoms as the first "filter" on the way to treatment. Several reasonable explanations for this "filter" can be suggested. Adolescents' and parents' ability to recognize their discomfort as mental health problems for which help might be sought might be limited. In line with this, many might consider symptoms reported on HSCL as normal conditions, and a part of the turmoil of adolescence. Stigma concerning mental health problems, and lack of trust in mental health services might prevent adolescents to seek help. Even though these numbers appear in the context of a free and relatively well developed health care system, limited availability of mental health services might also contribute to these low rates.

The hierarchical organization of the help-seeking pathway addresses the degree of specialization of health services contacted. We arranged health services hierarchically according to their specialization in mental health. In line with previous research [20] the MD (including GP) see a large proportion (44%) of adolescents seeking help for mental health problems. According to Norwegian public health policies (being similar to the UK system), adolescents are supposed to *first *consult their GP in presence of mental problems in need of treatment. GPs subscribe medication and provide consultations. Referrals to more specialized services are supposed to be the second option. In Norway, consulting psychologists and psychiatrists in private practice is publicly sponsored. Contact with these practitioners must be made through the GP if the expenses are to be covered by the public health care system. The fact that nearly half of the help-seeking adolescents do not get further than the MD might be explained partly as some getting help from the MD, partly some being referred but being on waiting lists, and partly some not having their concerns recognized by the MD.

Two of our findings might indicate that GPs have only a marginal role in treating anxiety and depression in adolescents. (1) The association between mental problems and help-seeking from GPs was strongly attenuated and marginally significant having adjusted for somatic and psychosomatic problems, which was not the case for contact with more specialized services like psychiatrists and psychologists. (2) GPs were the most specialized contact for about half of adolescents reporting help-seeking for mental health problems last 12 months. However, we have no information as to the clinical content of this contact. This figure is therefore certainly an overestimation of GPs role in treating mental health problems. These two indications of the possible limited role of GPs in treating anxiety and depression in adolescents are in contrast with the public health policy where GPs are supposed to detect and treat the majority of adolescents with mental health problems.

These results might suggest that adolescents with mental health problems seek help from areas of the health care system that are not specifically resourced to treat mental health problems. In line with our suggestion above, this might be explained by adolescents' limited capacity to recognize and understand their distress as anxiety and depression. These results are in line with Zwaanswijk et al's [8] suggestion that there is limited knowledge among adolescents on mental health questions. From a public health perspective, providing information about mental health issues would possibly increase awareness of mental health problems among adolescents. This might in turn lead to more adolescents seeking appropriate help for their problems [21].

It must also be considered whether the adolescents themselves or rather adults like parents, teachers, or others take the initiative to seek help. Fifteen and sixteen year olds are in between still being under protection of parents, and taking responsibility on their own. The broad array of health services contacted might be interpreted both as a result of concerned parents not knowing what's wrong with their children, and adolescents' not knowing where to go. In any case, the importance of this finding is that health professionals throughout the health care system are in contact with a relatively large number of adolescents with symptoms of anxiety and depression.

## Conclusion

Our study supports previous findings suggesting that only a minority of adolescents with mental health problems seeks help, and elaborate on these through the dose-response relation found between symptom load and help-seeking. We do not conclude that all adolescents with case-level mental health problems are in need of treatment. However, concerning the 99^th ^percentile, we think that claiming treatment need is less controversial. When the Norwegian context where mental health services are relatively available and free of charge is taken into account, help-seeking in adolescents with the highest symptom-loads is low. Approximately half of the help seekers achieved contact with the GP, and fewer reached a psychologist/psychiatrist.

However, our results might suggest that GPs play a relatively marginal role in detection and treatment of mental health problems among adolescents. Our results also suggest that adolescents' recognition of mental health problems or intention to seek help for these are the major "filters" restricting treatment.

Longitudinal population-based studies are needed to examine if and to what extent the prognosis of adolescents with mental health problems depends on treatment.

## Competing interests

The author(s) declare that they have no competing interests.

## Authors' contributions

HDZ participated in the literature review, in developing hypotheses and statistical analyses, and drafted the manuscript. KR participated in literature review, in generating hypotheses, statistical analyses and preparation of the first draft of the manuscript. AM participated in the development of hypotheses, the statistical analyses, and supervised the drafting of the manuscript. All authors have read and approved the final manuscript.

## Pre-publication history

The pre-publication history for this paper can be accessed here:



## Figures and Tables

**Table 1:** Level of specialization in health services reached

	Those seeking help for mental health problems^1 ^% (n)
No services	4.1 (30)
Nurse	3.3 (24)
MD	44 (320)
PPT/fam. Counseling	8.1 (59)
Psychologist/psychiatrist	39.8 (289)

^1 ^N = 722

Numbers represent percentages (n) of adolescents with a mental health problem for which help has been sought reaching each level of specialized services, but no further in the hierarchy.

**Table 2:** Health services contacted by adolescents with symptoms of anxiety and depression

		**Unadjusted effects**		**Adjusted for Psysom^1 ^and Som^2^**	
	N	OR	CI (95%)	OR	CI (95%)

Psychologist or psychiatrist	585	1.98	1.86–2.10	1.85	1.72–1.99
Municipal social service agency	210	1.65	1.50–1.80	1.53	1.37–1.72
Family counselling	459	1.57	1.46–1.68	1.48	1.36–1.60
Youth health clinic	1472	1.56	1.48–1.62	1.40	1.33–1.48
School health services	2706	1.43	1.37–1.49	1.28	1.22–1.43
Adolescent Pedagogical Psychological Service	519	1.38	1.29–1.49	1.28	1.18–1.39
Alternative therapy	856	1.42	1.35–1.50	1.20	1.12–1.28
Other medical specialist	1167	1.35	1.28–1.40	1.20	1.13–1.27
Hospital inpatient	724	1.34	1.26–1.43	1.15	1.07–1.23
GP	5983	1.34	1.28–1.39	1.11	1.06–1.16
Emergency ward or outpatient clinic	3355	1.26	1.12–1.13	1.10	1.05–1.15
Physiotherapist	1185	1.26	1.19–1.33	1.01^a^	.95–1.08

^1^Psychosomatic symptoms ^2^Somatic symptoms ^a^Non-significant. All other coefficients p < .001. Results of logistic regression showing increased OR for adolescents with symptoms of anxiety and depression in contact with different health services with and without adjustment for somatic and psychosomatic symptoms.
